# Global influenza epidemiology after 2020: patterns of circulation, epidemic timing and duration, and implications for vaccination strategies

**DOI:** 10.2807/1560-7917.ES.2026.31.21.2500743

**Published:** 2026-05-28

**Authors:** Marco Del Riccio, Saverio Caini

**Affiliations:** 1 Department of Health Sciences, University of Florence, Florence, Italy; 2 University of Florence, Florence, Italy

**Keywords:** influenza epidemic timing, viral ecology, trivalent vaccines, respiratory surveillance, programme planning, COVID-19 pandemic

## Abstract

**INTRODUCTION:**

There remains uncertainty whether global influenza seasonality, viral dynamics and epidemic duration have re-established after 2021.

**AIM:**

We describe global circulation patterns of influenza viruses from 2021 to 2025 and discuss implications for prevention and surveillance.

**METHODS:**

We analysed World Health Organization (WHO) FluNet sentinel and non-sentinel/not-defined virological data from week 1/2021 to 26/2025, stratifying by latitude, region and season. We calculated influenza positivity rate, proportion of virus (sub)types, typical peak timing and duration of influenza epidemics (applying the 75% annual average percentage method).

**RESULTS:**

Sentinel surveillance in 120 countries reported 500,870 detections in this period; positivity rate rose globally from 3.0% in 2021 to 23.7% in 2024. Type A viruses caused over two-thirds of cases, with variability across WHO Regions and seasons. Among A subtypes, A(H3N2) dominated in 2021/22 and A(H1N1)pdm09 in 2023/24, while all but three influenza B cases were B/Victoria. Epidemic peaks typically occurred from December to March and May to August in northern and southern hemispheres countries, respectively, while tropical countries showed highly heterogeneous timing. The median epidemic duration was ca 10 weeks above 30° north, and it varied between 15 and 30 weeks at more southern latitudes. Non-sentinel/not-defined feeds showed stronger A-skew and lower characterisation.

**CONCLUSION:**

Influenza virus circulation shows convergence toward seasonal architectures described before the COVID-19 pandemic, although changes in lineage ecology and epidemic duration persist. Our results confirm the need for latitude-tailored vaccination schedules, consolidation of trivalent vaccines, and strengthened surveillance to better anticipate changes in influenza circulation and support preparedness.

**Key public health message**

**What did you want to address in this study and why?**

After the unprecedented disruption of influenza circulation in 2020 and 2021, it was unclear whether global seasonality and subtype dynamics would return to pre-COVID-19 patterns, or whether new equilibria had emerged with consequences for vaccination planning. To support global policy, we analysed data from 2021 to 2025 to provide a picture of virus circulation, peak timing and epidemic duration indifferent geographical areas.

**What have we learnt from this study?**

Different types of influenza viruses predominated across seasons, with marked changes over time. Seasonal peaks re-aligned with latitude in temperate regions, but timing in the tropics remained heterogeneous. Epidemics were short in the northern hemisphere and longer in the temperate south, a difference that persisted despite pandemic disruption.

**What are the implications of your findings for public health?**

We show that influenza seasonality is resynchronising after the pandemic shock, but with durable changes in epidemic duration and the types of circulating viruses. This confirms the continued need for latitude-specific vaccination calendars, refines recommendations for the southern hemisphere and tropical countries, and supports the transition to trivalent vaccines. Local influenza patterns should guide vaccination timing and planning.

## Introduction

Seasonal influenza exhibits recurrent yet heterogeneous epidemiological patterns across climates and geographies. In temperate regions, epidemics usually show winter peaks with strong seasonal forcing, whereas tropical and subtropical settings display more variable timing - single peaks outside winter, multiple peaks, or near year‑round activity—modulated by local climatic and ecological drivers [1-5]. Key descriptors of these dynamics include the timing of epidemic onset and peak and the duration of epidemics, each of which has direct relevance for the synchronisation of preparedness activities and vaccination campaigns in diverse settings. Global situational awareness relies on the World Health Organization’s (WHO) Global Influenza Surveillance and Response System (GISRS) and the associated FluNet platform, which collate weekly virological detections from a network of national influenza centres and reference laboratories [6,7]. These data underpin the biannual WHO consultations on vaccine composition, aligning production and deployment with expected activity windows in the northern and southern hemispheres.

Before 2020, and in particular after the A(H1N1)pdm09 influenza pandemic, a broad evidence base had consolidated several features of influenza seasonality: winter peaking and marked seasonality in temperate zones; substantial variability in timing and intensity in the intertropical belt (ITB); and latitudinal gradients in periodicity and predictability [1-5]. Within this landscape, Zanobini et al. provided a comprehensive GISRS/FluNet‑based baseline for the period 2010 to 2020, reporting predominance of influenza A viruses across most seasons and regions, winter peaks at higher latitudes, greater variability nearer the equator, and typically shorter epidemics at high absolute latitudes [8].

The control measures put in place during the COVID-19 pandemic produced a marked disruption in these patterns: during 2020 and 2021, many countries observed unprecedented declines in influenza circulation, altered synchrony between regions and atypical seasonality, probably influenced by non‑pharmaceutical interventions, changes in healthcare seeking and testing, and reduced international mobility [9,10]. Subsequent analyses have suggested that the interruption of exposure may have reshaped population immunity and viral ecology, with potential consequences for the timing, duration and lineage dynamics of future influenza seasons [11-13]. These uncertainties directly affect operational questions: when to vaccinate in low‑latitude settings with variable peak timing; how to anticipate the duration and intensity of epidemics under post‑pandemic conditions; and whether pre‑2020 assumptions about inter‑regional synchrony remain valid.

Despite extensive pre‑pandemic mapping of global patterns, consolidated post‑2020 assessments using harmonised metrics remain limited. Specific gaps include: (i) updated global distributions of influenza virus types, subtypes and lineages across WHO Regions and latitude bands; (ii) standardised quantification of peak timing and epidemic duration across climatic zones; and (iii) the implications of these metrics for the scheduling of vaccine programmes, particularly where waning of protection within a season is a consideration [14]. Addressing these gaps is essential to refine recommendations for temperate and tropical settings alike.

This study provides a global update extending the observation window beyond 2020 and leveraging GISRS/FluNet data to examine whether, and how, the viral circulation of influenza viruses and the seasonality of epidemics has changed in the post‑COVID-19 period. By applying the same underlying data source and a highly comparable analytical framework as used in pre-pandemic global analyses of GISRS/FluNet data [8], this study allows a qualitative comparison across periods while focusing on the early post-pandemic seasons. Specifically, we aimed to (i) characterise the contemporary distribution of influenza virus types and subtypes/lineages by region and latitude band and (ii) describe seasonality using harmonised indicators of timing and duration. The goal was to generate policy-relevant evidence that may inform influenza vaccination scheduling and surveillance planning across different epidemiological settings.

## Methods

This was a retrospective, descriptive surveillance study based on routinely collected virological data from the WHO GISRS, aimed at characterising post-pandemic patterns of influenza virus circulation, epidemic timing and duration at the global level.

### Data sources definitions

Weekly counts of processed respiratory specimens and influenza detections (by virus type, subtype and lineage) from week 1/2021 to week 26/2025 were downloaded from the WHO FluNet database (https://www.who.int/tools/flunet) on 15 July 2025 [7]. Each country was assigned to the northern hemisphere (NH), the southern hemisphere (SH) or the ITB, depending on whether its geographic centroid was located north of the Tropic of Cancer, south of the Tropic of Capricorn, or between the two tropics, respectively. Because the timing of influenza activity differs across latitudes, for the purposes of this analysis we defined a ‘season’ (i.e. an operational time window used for analysis that may not fully coincide with the conventional epidemiological influenza season or epidemics) as the period from week 27 (beginning of July) of a given year to week 26 (end of June) of the following year in NH countries, or as the calendar year in ITB and SH countries. As a consequence of this definition, analysing surveillance data from week 1/2021 to week 26/2025 meant that influenza surveillance data from up to four full ‘seasons’ could be available for countries in both the NH (from 2021–22 to 2024–25) and the ITB and SH (from 2021 to 2024). Of note, surveillance data for the weeks 1–26/2021 were not used for NH countries as they would correspond to only half a season in that geographical area; for the same reason, data for weeks 1-26/2025 were not used for ITB and SH countries. For brevity, in what follows, we will use ‘season 2021’ to refer to both the 2021 season in the ITB and SH, and to the 2021–22 season in the NH.

Each country can contribute data to the FluNet database, which are classified into one of three different categories (Box):

Box **FluNet data categories**

- Non-sentinel: Data obtained from non-sentinel systems as indicated by the reporting country. Data reported in this category may include outbreak investigation, universal testing, testing at point of care or other systems apart from sentinel surveillance.
- Sentinel: Data obtained from sentinel surveillance as indicated by the reporting country. Sentinel surveillance systems collect high-quality data in a timely manner systematically and routinely from sentinel surveillance sites representative of the population under surveillance.
- Type not defined: Source of data not indicated by the reporting country neither as sentinel nor as non-sentinel surveillance. These data may include sentinel or non-sentinel surveillance sources or both.

Category description reproduced from [7].

In each season, a country could contribute data of one type (sentinel, non-sentinel or not-defined) or, less frequently, more than one type (typically sentinel and non-sentinel). Because surveillance system design may affect some indicators, we refrained from merging data of different types. However, previous analyses found only minor differences in epidemic timing using surveillance data from sentinel and non-sentinel sources: in 23 countries with parallel data, peak timing differed by < 1 week in 72% and by < 2 weeks in 91% of cases, with no consistent bias in either direction [13]. To maximise completeness while preserving consistency, we therefore analysed sentinel and non-sentinel/not-defined data separately; in the rare cases where both non-sentinel and not-defined data were available for the same season, the former source was used.

### Statistical analysis

The unit of analysis was the ‘country-season’, defined as data for a ‘season’ (as per our definition) from a given country. Country-seasons with data available for less than 30 weeks were removed from the dataset, regardless of the source of data (sentinel, non-sentinel or not-defined) and the number of processed respiratory specimens and reported influenza detections, to reduce the risk that the influenza epidemic in that season was not captured in its entirety (from onset to end). Moreover, we included only country-seasons with ≥ 50 influenza detections to ensure robustness. We applied this threshold separately to each surveillance data source (sentinel, non-sentinel or not-defined), and not to pooled country-level data across sources. The analytical approach and key metrics used in this study (including peak estimation and the 75% average annual percentage method for epidemic duration) are consistent with those applied in our previous global analysis of GISRS/FluNet data for 2010 to 2020, in order to support comparability of timing and duration indicators across periods [8].

We calculated in each country-season the percentage of all influenza detections that were caused by either virus type (A or B). We then determined its median value and the proportion of country-seasons in which influenza A was responsible for ≥ 80%, ≥ 50 to 80%, ≥ 20 to 50% or < 20% of all influenza cases. This was done in the entire dataset, separately in countries located in the same geographical area (NH, ITB or SH) or in the same WHO Region (Africa, Eastern Mediterranean, European, Americas, South-East Asia and Western Pacific), and by season (from 2021 to 2024). These calculations were repeated for each type A virus subtype (H3N2, H1N1pdm09 or other/unsubtyped) and type B virus lineage (Victoria or uncharacterised – influenza B/Yamagata detections were so rare that we merged them with uncharacterised ones), in the whole dataset and separately in each of the four seasons included in the analysis. Analyses were performed separately for sentinel and for non-sentinel/not-defined surveillance data; country-level and latitude-band summaries were computed within each type of data source, without pooling observations across different streams.

Next, we calculated the influenza positivity rate, defined as the ratio between the number of detections and the number of processed specimens, and reported it separately by virus type, subtype and lineage, in the whole dataset and stratifying according to geographical area, WHO Region and season. Unlike the other analyses described in this section, the positivity rate was not calculated for non-sentinel/not-defined data, as the latter may consist of specimens collected in various and often not well-defined settings, thus making the positivity rate difficult to interpret.

We then determined the typical timing of the influenza epidemic peak by using the EPIPOI software (https://www.epipoi.info). This software works by first detrending each country-specific time-series by means of second-degree polynomials and then proceeds to determine the periodic annual function (PAF) by summing up the annual, semi-annual and quarterly harmonics obtained via Fourier decomposition [15]. The timing of the peak of the PAF is reported in what follows as the typical timing of the peak of influenza epidemics in each country.

The duration of the influenza epidemic in each country-season was calculated using the average annual percentage method (AAP) with a 75% threshold, which consists of identifying the shortest period of consecutive weeks accounting for ≥ 75% of all influenza detections in that country-season. Epidemic onset and end were defined as the first and last week of this period, respectively, and duration as the number of weeks between onset and end.

We then calculated the median value of the duration of influenza epidemics in each 10-degree latitude bands, i.e. among all available seasons from countries with latitude between 40° and 30° south, 30° and 20° south etc., up to 60° to 70° north.

The analyses were conducted using Stata version 17 (Stata Corp, College Station, US) and EPIPOI (https://www.epipoi.info).

## Results

### Influenza virus circulation and positivity rates

Before applying exclusion criteria, the dataset comprised sentinel data from 149 countries (502,240 influenza detections) and non-sentinel/not-defined data from 150 countries (3,940,499 detections). After exclusions, 120 countries provided a total of 369 country-seasons with sentinel surveillance data, encompassing over 4 million respiratory specimens and 500,870 influenza detections, with an overall 12.2% positivity rate (Table 1). In the non-sentinel/not-defined stream, 121 countries contributed 3,930,762 detections. Overall, 29 countries were excluded from each data source, accounting for 1,370 detections in sentinel data (0.27%) and 9,737 detections in non-sentinel/not-defined data (0.25%). Type A and B influenza viruses caused 80.9% and 19.1% of all influenza detections globally, with a positivity rate of 9.9% and 2.3%, respectively. More in detail, the median proportion of influenza detections caused by type A viruses was 76.4%, and the proportion of all influenza cases that were caused by type A viruses was ≥ 80% in 44.7% of country-seasons, ≥ 50% and < 80% in 46.6% of country-seasons, ≥ 20% and < 50% in 7.6% of country-seasons, and < 20% in only 1.1% of country-seasons. 

**Table 1:** Global circulation of type A and B influenza viruses according to latitude, WHO Region, and season, sentinel surveillance data, 2021–2025 (n = 500,870)

	n country-seasons (≥ 50 cases)	Influenza cases		Influenza type A			Influenza type B			Median cases/ season	Median % type A	Season with % type A			
		n	% positivity rate	n	%	% positivity rate	n	%	% positivity rate			≥ 80%	≥ 50% to < 80%	≥ 20% to < 50%	< 20%
**Geographical area**															
Northern hemisphere	225	416,244	12.5	344,584	82.8	10.4	71,660	17.2	2.2	384	78.3	109	98	17	1
Inter-tropical belt	130	68,957	10.4	49,104	71.2	7.4	19,853	28.8	3.0	346	74.0	50	67	10	3
Southern hemisphere	14	15,669	14.3	11,695	74.6	10.7	3,974	25.4	3.6	1,012	76.9	6	7	1	0
**WHO Region**															
African Region	48	21,297	11.1	16,055	75.4	8.4	5,242	24.6	2.7	308	74.0	17	30	1	0
Eastern Mediterranean Region	46	38,848	11.0	27,805	71.6	7.9	11,043	28.4	3.1	317	75.8	18	24	4	0
European Region	160	102,615	17.2	75,974	74.0	12.7	26,641	26.0	4.5	313	81.0	82	63	14	1
Region of the Americas	71	282,308	11.0	243,000	86.1	9.5	39,308	13.9	1.5	497	74.5	29	31	8	3
South-East Asia Region	21	17,546	9.8	13,180	75.1	7.4	4,366	24.9	2.4	548	76.1	7	14	0	0
Western Pacific Region	23	38,256	17.9	29,369	76.8	13.7	8,887	23.2	4.2	635	85.1	12	10	1	0
**Season**															
2021	48	40,760	3.0	39,030	95.8	2.8	1,730	4.2	0.1	176	99.1	44	2	1	1
2022	102	108,246	10.9	87,156	80.5	8.7	21,090	19.5	2.1	410	74.0	34	59	8	1
2023	114	136,960	16.8	102,917	75.1	12.6	34,043	24.9	4.2	367	81.5	59	46	7	2
2024	105	214,904	23.7	176,280	82.0	19.5	38,624	18.0	4.3	518	70.4	28	65	12	0
**Total**	**369**	**500,870**	**12.2**	**405,383**	**80.9**	**9.9**	**95,487**	**19.1**	**2.3**	**375**	**76.4**	**165 (44.7%)**	**172 (46.6%)**	**28 (7.6%)**	**4 (1.1%)**

WHO: World Health Organization.

Source: WHO FluNet database. Only country-seasons with ≥ 50 reported influenza detections are included.

These figures varied somewhat when stratifying by latitude-defined geographical area (Table 1): the overall positivity rate was higher in countries in the SH (14.3%) and NH (12.5%), and lower in countries in the ITB (10.4%). However, the median proportion of influenza detections caused by type A viruses differed only moderately by geographical area, ranging from 78.2% in the NH to 74.0% in the ITB. Greater variability emerged across WHO Regions (Table 1): the positivity rate ranged from < 10% in the South-East Asia Region to > 17% in the European and Western Pacific Regions, and type A viruses caused a median proportion of all influenza cases ranging from < 75% in the Africa Region and the Region of the Americas to > 85% in the Western Pacific Region. There were notable differences between seasons as well (Table 1). The overall influenza positivity rate rose steadily from 3.0% in 2021 to 23.7% in 2024, and the proportion of influenza cases accounted for by type A viruses ranged between 99.1% in 2021 and 70.4% in 2024 (median values). 

Country-level results for non-sentinel/not-defined surveillance data are provided in the Supplement. Considering those data, the proportion of influenza cases caused by type A viruses was consistently higher than among sentinel surveillance data, e.g. the median proportion calculated across all country-seasons was 83.9% vs. 76.4%. The wide fluctuation between seasons was confirmed, with influenza A having a much larger impact in 2021 and 2023 than in 2022 and 2024.

The proportion of influenza detections caused by each type A virus subtype and type B virus lineage over the total number of influenza cases detected by sentinel surveillance systems globally in each season is reported in Table 2. The influenza A(H3N2) virus subtype was the most prevalent in 2021 (76.5% of all influenza detections globally) and 2022 (51.5%), while the influenza A(H1N1)pdm09 virus subtype caused the largest share of influenza cases in 2023 (40.2%, vs 24.8% for H3N2) and 2024 (37.2%, vs. 33.6% for H3N2). The proportion of influenza cases caused by unsubtyped type A viruses (or subtypes other than H1N1 and H3N2) ranged between 10.2% in 2024 and 17.1% in 2021. Concerning type B influenza, 59.8% of total detections were characterised during the study period: all were found to belong to the Victoria lineage, except for three B/Yamagata detections (Table 2). 

**Table 2:** Global circulation of influenza virus types, subtypes and lineages by season, sentinel surveillance data, 2021–2025 (n = 500,870)

Season	n country-seasons (≥ 50 cases)	Influenza cases		A(H3N2)			A(H1N1)			Type A other or unsubtyped			B Victoria			B uncharacterised^a^		
		n	% positivity rate	n	%	% positivity rate	n	%	% positivity rate	n	%	% positivity rate	n	%	% positivity rate	n	%	% positivity rate
2021	48	40,760	3.0	31,172	76.5	2.3	878	2.2	0.1	6,978	17.1	0.5	680	1.7	0.0	1,050	2.6	0.1
2022	102	108,246	10.9	55,747	51.5	5.6	18,848	17.4	1.9	12,554	11.6	1.3	10,907	10.1	1.1	10,183	9.4	1.0
2023	114	136,960	16.8	33,978	24.8	4.2	55,019	40.2	6.8	13,914	10.2	1.7	26,376	19.3	3.2	7,667	5.6	0.9
2024	105	214,904	23.7	72,176	33.6	8.0	79,851	37.2	8.8	24,152	11.2	2.7	19,130	8.9	2.1	19,494	9.1	2.2
**Total**	**369**	**500,870**	**12.2**	**193,073**	**38.5**	**4.7**	**154,596**	**30.9**	**3.8**	**57,598**	**11.5**	**1.4**	**57,093**	**11.4**	**1.4**	**38,394**	**7.7**	**0.9**

WHO: World Health Organization.

^a^ This also includes three influenza B Yamagata detections.

Source: WHO FluNet database. Only country-seasons with ≥ 50 reported influenza detections were included.

In non-sentinel/not-defined data, the virus subtype/lineage was determined for a much smaller proportion of influenza detections, namely 28.4% and 25.5% of influenza A and B cases, respectively. The H3N2 virus subtype was responsible for the majority of influenza A cases in all seasons except 2024, while among influenza B cases, all but six cases of those that were characterised belonged to the B/Victoria lineage. The detailed country-level results for non-sentinel surveillance data can be accessed in the Supplement.

### Timing of influenza epidemic peaks

**Figure 1:**
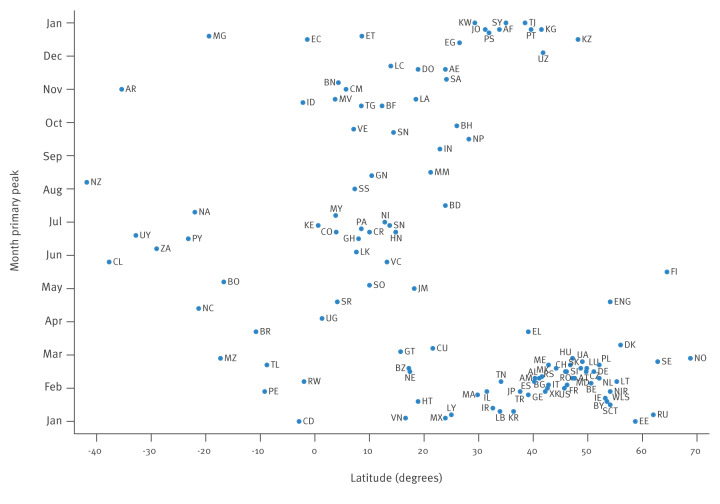
Typical timing of the primary peak of influenza detections by country, against the latitude of the country centroid, sentinel surveillance data, 2021–2025 (n = 500,870)

The typical timing of the influenza epidemic peak against the country latitude is depicted in Figure 1 for sentinel surveillance data. For NH countries, the typical timing of the epidemic peak occurred between the second half of December and the first half of March, with only a few exceptions, while for SH countries, the epidemic peak typically took place between the end of May and the beginning of August, again with only a limited number of exceptions. In contrast, the ITB encompassed countries whose epidemic peak could occur in practically any month of the year, although with clusters between June and August (Figure 1) between October and November.

The abbreviations in the Figure correspond to country labels in the form of ISO 3166-1 alpha-3 codes (with a few non-standard exceptions). For a list defining those labels, please see Supplementary Table S3.

Source: WHO FluNet database.

### Onset, end and duration of influenza epidemics

**Figure 2:**
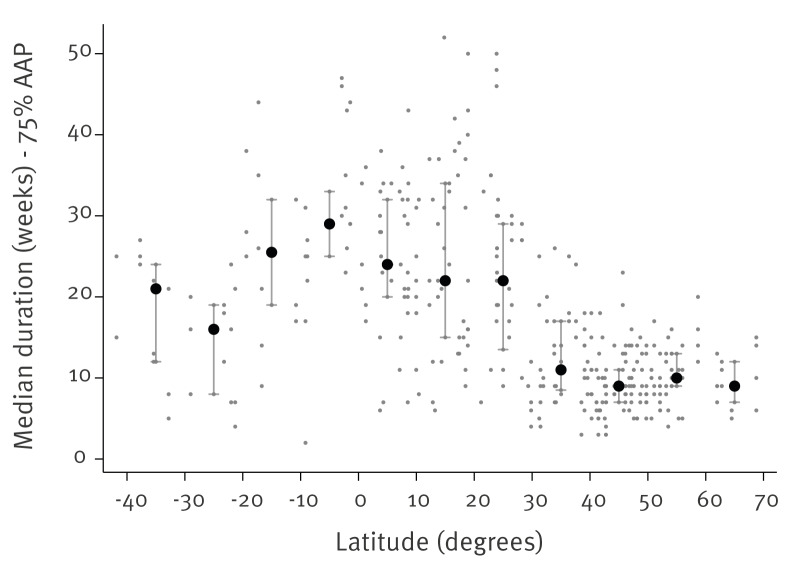
Duration of influenza epidemics, by latitude, sentinel surveillance data, 2021–2025 (n = 500,870)

The median duration of influenza epidemics against the country’s latitude is depicted in Figure 2 for sentinel surveillance data. In countries north of 30° north latitude, the vast majority of country-seasons had influenza epidemics lasting less than 20 weeks, with a median value (calculated by 10-degree latitude bands) of around 10 weeks. For latitudes south of 30° north, there was much greater variability, with country-seasons in which the influenza epidemic could be concentrated in very few weeks (< 10) or extend throughout the year (over 40 weeks). Of note, the duration of influenza epidemics was longer in SH countries (median value 21 weeks for countries lying between 30° and 40° south, and 16 weeks for those between 20° and 30° south) compared with NH countries. This picture was largely confirmed when examining non-sentinel/not-defined surveillance data; the country-level results for non-sentinel surveillance data can be accessed in the Supplement.

AAP: annual percentage.

Duration is calculated according to the 75% AAP method. The small grey dots represent individual country-seasons, while the large black dot represent the median values by 10-degree latitude bands (from 40–30° south to 60–70° north); vertical bars indicate the 25th and 75th percentiles (interquartile range) within each latitude band.

Source: WHO FluNet database.

Epidemic onset also varied by latitude. In countries north of 30° north latitude, onset values were concentrated within a relatively narrow range around the year boundary, with median onset by 10-degree latitude bands occurring predominantly in late-year/early-year weeks and with comparatively smaller interquartile ranges at higher northern latitudes. South of 30° north, onset values were more dispersed, with the widest spread observed in the ITB. Overall patterns were consistent in sentinel and non-sentinel/not-defined analyses, although dispersion was greater in the latter. Country-level results are provided in the Supplement.

## Discussion

The present analysis offers an integrated picture of influenza circulation during the first four post‑COVID-19 seasons captured by GISRS/FluNet and suggests a substantive, though not complete, convergence toward canonical seasonal architectures. Across 369 sentinel country‑seasons, more than 4 million respiratory specimens were processed, yielding 500,870 influenza detections. Based on these, influenza A predominated (median 76.4% of detections), with broadly similar A/B proportions across latitude bands but notable heterogeneity across WHO Regions. Positivity increased from 3.0% in 2021 to 23.7% in 2024, suggesting a progressive restoration of influenza activity after the disruption in 2020 and 2021; the observed shifts between influenza A(H3N2) and A(H1N1)pdm09 are consistent with subtype fluctuations already described before the COVID-19 pandemic. Characterised influenza type B detections were nearly exclusively B/Victoria, with only sporadic putative B/Yamagata. Peak timing again aligned with latitude (December–March in temperate northern settings; May–August in temperate southern settings); the ITB remained heterogeneous, with mid-year clusters and a secondary October–November cluster in sentinel data. 

Use of the most up-to-date vaccine formulation before the expected period of influenza activity is relevant across settings. In ITB countries, however, the greater heterogeneity in epidemic timing makes country-specific surveillance particularly important for guiding vaccination schedules. More generally, vaccination campaigns are likely to be most effective when implemented close to the expected start of influenza circulation and when achieving high coverage early in the season, rather than being prolonged over several months. The onset patterns refine this message by showing that, in settings where seasonal activity is tightly synchronised, the start of the main epidemic period is sufficiently concentrated to make early, high-coverage delivery the dominant determinant of impact. By contrast, where onset is more dispersed, most notably in the ITB, optimising lead time becomes less about choosing a single best start date and more about using current local surveillance to time delivery so that protection overlaps the locally relevant rise in activity. 

Epidemic duration was short above 30° north (median ca 10 weeks by latitude bands) but longer and more variable in temperate SH countries (median ca 16–21 weeks).These patterns were broadly consistent in non‑sentinel feeds, although the latter showed a higher A‑skew (a systematic predominance of influenza A over influenza B detections) and substantially lower rates of subtyping and lineage characterisation. The longer median duration of influenza epidemics observed in temperate SH countries compared with the NH may reflect differences in climatic forcing and seasonal amplitude, resulting in less abrupt transitions into and out of the influenza season. In addition, variations in population mobility, school calendars and surveillance sensitivity may contribute to a more prolonged detection of influenza activity, even when peak intensity is comparable [1,4].

Placed against pre‑pandemic baselines, these findings indicate that the pandemic shock seems to have subsided with respect to peak timing, but that the virological landscape and timing of onset and peak have not completely reverted to the situation between 2010 and 2020. The latitudinal gradient in timing we document mirrors the well‑described winter peaks at higher absolute latitudes and heterogeneous activity nearer the equator [1,3-5,8]. The marked contraction of epidemic duration in the temperate north and the longer, more variable seasons in parts of the temperate south are directionally consistent with pre‑2020 evidence [5,8], yet the magnitude of the south–north asymmetry and the persistence of ITB bimodality underscore the importance of local calibration. The steady rise in positivity rates and the re‑emergence of familiar peak windows suggest a progressive re-synchronisation after the near‑absence of circulation and irregular timing documented in 2020 and 2021 [9,10], but this apparent return in epidemic timing does not necessarily imply a full restoration of the pre-pandemic virological landscape, particularly in light of the near absence of confirmed B/Yamagata detections. In the absence of a valid counterfactual scenario, however, it remains uncertain whether similar changes in subtype and lineage composition would have occurred without the COVID-19 pandemic. Overall, the timing and duration patterns observed during the period 2021 to 2025 are consistent with those described in global analyses before 2020, suggesting convergence toward established seasonal architectures rather than a persistently disrupted post-pandemic pattern [8]. The subtype and lineage patterns are both epidemiologically relevant and programmatically important. The observed shifts between influenza A(H3N2) and A(H1N1)pdm09 should therefore be interpreted as part of the known multi-year variability in influenza A subtype predominance, while recognising that the short post-pandemic observation window limits inference on the mechanisms underlying these patterns [8]. The near absence of confirmed influenza B/Yamagata, despite very large denominators and extensive geographical coverage, and the overwhelming predominance of influenza B/Victoria support the growing consensus that influenza B/Yamagata is probably extinct in human circulation [12,16]. This shift has already been reflected in policy: The WHO recommendations for the NH 2024/25 and 2025/26 seasons as well as 2026 SH season specified trivalent formulations [17,18], and regulators in the United States and European Union operationalised trivalent-only vaccines starting in 2024/25 [19-21]. This lineage loss has already altered the risk landscape and provides a coherent explanation for the narrower spread of A/B proportions across regions in our dataset.

Three main operational implications follow. Firstly, preparedness and clinical surge planning can again be anchored to latitude‑informed timing, but with attention to duration asymmetries: temperate northern countries can plan for short, concentrated seasons, while temperate southern countries should anticipate longer and more variable risk periods. Secondly, the ITB remains inherently heterogeneous: reliance on national or subnational surveillance to tune the calendar is therefore indispensable, and phased interventions may be warranted in settings with multiple peaks. Thirdly, the surveillance value chain requires targeted upgrades precisely where the burden is most severe: non‑sentinel feeds captured a higher proportion of influenza A but contributed far less subtype/lineage characterisation, limiting situational awareness and the policy utility of hospital‑proximate data. Minimum subtyping quotas for diagnoses of severe acute respiratory infections, routine month‑stratified sequencing of a defined fraction of hospital detections, and harmonised metadata (care setting, age, comorbidity, vaccination status) would materially improve inference without imposing unrealistic laboratory burdens. These steps are aligned with the GISRS mandate and global influenza strategy and would directly feed into WHO vaccine composition deliberations.

Vaccination policy needs to consider both when influenza circulates and which viruses are circulating: with seasonality largely re‑established in temperate zones, immunisation windows can keep conventional schedules, provided doses are administered close enough to the expected onset to avoid substantial within‑season waning — most pronounced against influenza A(H3N2) [14]. In the NH, this generally supports early autumn initiation for most adults, with vaccination continuously offered while viruses circulate; in the temperate SH, earlier starts or extended vaccine availability may be justified by the longer median durations we document. In the ITB, a single annual campaign is frequently misaligned with local peaks; tailored calendars and, where logistics permit, phased or biannual targeting of high‑risk groups (older adults, people with chronic conditions, pregnant persons) are prudent. The virological context also argues for consolidating trivalent formulations that exclude influenza B/Yamagata. Continued high-quality genetic characterisation remains critical to detect any unexpected lineage re-emergence and to track antigenic drift in influenza A(H1N1)pdm09 and A(H3N2) for timely vaccine virus updates.

This study faces a number of limitations. Tight comparison with the 2010 to 2020 baseline [8] requires caution, because the four-season observation window increases the influence of outliers on country-level estimates and limits the interpretation of subtype and lineage distributions, which may be affected by year-to-year fluctuations and incomplete characterisation. Duration metrics based on the 75% AAP mitigate biases from multi-peak seasons, whereas peak timing is more sensitive to irregular waves and sparse early-season testing. Surveillance heterogeneity across countries, together with differences between sentinel and non-sentinel streams — often skewed toward severe or cases diagnosed in hospital settings — somewhat limit comparability and explain lower characterisation rates and a stronger A-skew in non-sentinel data. In addition, in the post-pandemic context, latitude- and Region-based analyses may reflect not only climatic and ecological drivers of influenza seasonality, but also residual heterogeneity related to the impact of COVID-19. This includes differences in the strain on health systems, in testing practices and capacity, in the pace of restoration of functional sentinel surveillance systems, in non-pharmaceutical interventions and vaccination strategies. These factors cannot be disentangled using aggregated GISRS/FluNet data and may contribute to the observed geographical contrasts. National-level analyses may also mask substantial subnational heterogeneity in influenza timing and intensity, particularly in large or climatically diverse countries, which could not be addressed using aggregated FluNet data. Incomplete subtyping and lineage attribution, particularly outside sentinel systems, may bias proportional estimates. Moreover, although influenza seasonality parameters are known to be influenced by virus subtype, antigenic novelty and vaccine effectiveness, the present analysis does not allow adjusting for these factors, nor directly assessing their relationship with epidemic timing, duration or clinical severity, as such information is not captured in aggregated FluNet surveillance data. Finally, our pragmatic thresholds of ≥ 50 detections and ≥ 30 reporting weeks improved the robustness of seasonality estimates but may exclude smaller, low-incidence or intermittently reporting country-seasons; we not formally assess sensitivity to alternative thresholds. These factors counsel caution in interpreting absolute values, although the relative patterns observed across systems remain robust.

Notwithstanding these caveats, the convergence of timing and duration signals across hemispheres and data streams, the consistent A predominance, and the virtual absence of influenza B/Yamagata constitute a coherent post‑pandemic picture. The novelty of this analysis lies less in reaffirming the latitudinal gradient itself than in quantifying the pace and extent of resynchronisation after the pandemic shock, documenting durable changes in lineage ecology with direct implications for vaccine policy, and highlighting residual south–north asymmetries in epidemic duration with tangible consequences for vaccination programmes logistics.

## Conclusions

Taken together, these findings call for maintaining and expanding sentinel networks with timely virological characterisation, closing the hospital‑proximate data gap through feasible subtyping and sequencing targets, and keeping vaccination calendars flexible by latitude and local history, explicitly accounting for waning so that protection overlaps the weeks of greatest risk. Additional seasons of surveillance will clarify whether the present equilibrium stabilises, capture the next phase of antigenic evolution, and refine vaccination schedules, particularly in the ITB, so that programmes deliver maximal population benefit.

## Data Availability

All data used in this study are publicly available through the World Health Organization’s Global Influenza Surveillance and Response System (GISRS). Weekly virological surveillance data can be accessed via the WHO FluNet platform at: https://www.who.int/tools/flunet. No additional data beyond those provided in the manuscript are available.

## Supplementary Data

336000 Supplement

## References

[1] Tamerius J Nelson MI Zhou SZ Viboud C Miller MA Alonso WJ. Global influenza seasonality: reconciling patterns across temperate and tropical regions. Environ Health Perspect. 2011;119(4):439-45. 10.1289/ehp.1002383 21097384 PMC3080923

[2] Azziz Baumgartner E Dao CN Nasreen S Bhuiyan MU Mah-E-Muneer S Al Mamun A Seasonality, timing, and climate drivers of influenza activity worldwide. J Infect Dis. 2012;206(6):838-46. 10.1093/infdis/jis467 22829641

[3] Li Y Reeves RM Wang X Bassat Q Brooks WA Cohen C Global patterns in monthly activity of influenza virus, respiratory syncytial virus, parainfluenza virus, and metapneumovirus: a systematic analysis. Lancet Glob Health. 2019;7(8):e1031-45. 10.1016/S2214-109X(19)30264-5 31303294

[4] Bloom-Feshbach K Alonso WJ Charu V Tamerius J Simonsen L Miller MA Latitudinal variations in seasonal activity of influenza and respiratory syncytial virus (RSV): a global comparative review. PLoS One. 2013;8(2):e54445. 10.1371/journal.pone.0054445 23457451 PMC3573019

[5] Caini S Andrade W Badur S Balmaseda A Barakat A Bella A Temporal Patterns of Influenza A and B in Tropical and Temperate Countries: What Are the Lessons for Influenza Vaccination? PLoS One. 2016;11(3):e0152310. 10.1371/journal.pone.0152310 27031105 PMC4816507

[6] World Health Organization (WHO). Global Influenza Surveillance and Response System (GISRS). Geneva: WHO. [Accessed: 1 Dec 2025]. Available from: https://www.who.int/initiatives/global-influenza-surveillance-and-response-system

[7] World Health Organization (WHO). Influenza laboratory surveillance information. Influenza virus detections reported to FluNet. Geneva: WHO. [Accessed: 1 Dec 2025]. Available from: https://app.powerbi.com/view?r=eyJrIjoiNjViM2Y4NjktMjJmMC00Y2NjLWFmOWQtODQ0NjZkNWM1YzNmIiwidCI6ImY2MTBjMGI3LWJkMjQtNGIzOS04MTBiLTNkYzI4MGFmYjU5MCIsImMiOjh9

[8] Zanobini P Bonaccorsi G Lorini C Haag M McGovern I Paget J Global patterns of seasonal influenza activity, duration of activity and virus (sub)type circulation from 2010 to 2020. Influenza Other Respir Viruses. 2022;16(4):696-706. 10.1111/irv.12969 35212157 PMC9178051

[9] Sullivan SG Carlson S Cheng AC Chilver MB Dwyer DE Irwin M Where has all the influenza gone? The impact of COVID-19 on the circulation of influenza and other respiratory viruses, Australia, March to September 2020. Euro Surveill. 2020;25(47):2001847. 10.2807/1560-7917.ES.2020.25.47.2001847 33243355 PMC7693168

[10] Karlsson EA Mook PAN Vandemaele K Fitzner J Hammond A Cozza V Review of global influenza circulation, late 2019 to 2020, and the impact of the COVID‑19 pandemic on influenza circulation. Wkly Epidemiol Rec. 2021;96:241-64. Available from: https://www.who.int/publications/i/item/who-wer-9625-241-264

[11] Dhanasekaran V Sullivan S Edwards KM Xie R Khvorov A Valkenburg SA Human seasonal influenza under COVID-19 and the potential consequences of influenza lineage elimination. Nat Commun. 2022;13(1):1721. 10.1038/s41467-022-29402-5 35361789 PMC8971476

[12] Caini S Meijer A Nunes MC Henaff L Zounon M Boudewijns B Probable extinction of influenza B/Yamagata and its public health implications: a systematic literature review and assessment of global surveillance databases. Lancet Microbe. 2024;5(8):100851. 10.1016/S2666-5247(24)00066-1 38729197

[13] Del Riccio M Caini S Bonaccorsi G Lorini C Paget J van der Velden K Global analysis of respiratory viral circulation and timing of epidemics in the pre-COVID-19 and COVID-19 pandemic eras, based on data from the Global Influenza Surveillance and Response System (GISRS). Int J Infect Dis. 2024;144:107052. 10.1016/j.ijid.2024.107052 38636684

[14] Young B Sadarangani S Jiang L Wilder-Smith A Chen MI-C. Duration of influenza vaccine effectiveness: a systematic review, meta-analysis, and meta-regression of test-negative design case-control studies. J Infect Dis. 2018;217(5):731-41. 10.1093/infdis/jix632 29220496

[15] Alonso WJ McCormick BJ. EPIPOI: a user-friendly analytical tool for the extraction and visualization of temporal parameters from epidemiological time series. BMC Public Health. 2012;12(1):982. 10.1186/1471-2458-12-982 23153033 PMC3527308

[16] Del Riccio M Nunes MC Cowling BJ Lina B McCauley JW Meijer A Post-disappearance scenarios: policy implications following the potential disappearance of B/Yamagata lineage influenza viruses. Euro Surveill. 2024;29(45):2400196. 10.2807/1560-7917.ES.2024.29.45.2400196 39512167 PMC11544721

[17] World Health Organization (WHO). Recommended composition of influenza virus vaccines for use in the 2024–2025 Northern Hemisphere influenza season. Geneva: WHO; 23 Feb 2024. Available from: https://www.who.int/publications/m/item/recommended-composition-of-influenza-virus-vaccines-for-use-in-the-2024-2025-northern-hemisphere-influenza-season

[18] World Health Organization (WHO). Recommended composition of influenza virus vaccines for use in the 2025–2026 Northern Hemisphere influenza season. Geneva: WHO; 28 Feb 2025. Available from: https://www.who.int/publications/m/item/recommended-composition-of-influenza-virus-vaccines-for-use-in-the-2025-2026-nh-influenza-season

[19] Grohskopf LA Ferdinands JM Blanton LH Broder KR Loehr J. Prevention and control of seasonal influenza with vaccines: Recommendations of the Advisory Committee on Immunization Practices-United States, 2024-25 influenza season. MMWR Recomm Rep. 2024;73(5):1-25. 10.15585/mmwr.rr7305a1 39197095 PMC11501009

[20] U.S. Food and Drug Administration (FDA). Use of trivalent influenza vaccines for the 2024–2025 U.S. influenza season. Silver Spring: FDA; 5 Mar 2024. Available from: https://www.fda.gov/vaccines-blood-biologics/lot-release/use-trivalent-influenza-vaccines-2024-2025-us-influenza-season

[21] European Medicines Agency (EMA). EU recommendations for 2024/2025 seasonal flu vaccine composition. Amsterdam: EMA; 26 Mar 2024 [updated 3 Jun 2024]. Available from: https://www.ema.europa.eu/en/news/eu-recommendations-2024-2025-seasonal-flu-vaccine-composition

