# Supplementary material for: Global influenza epidemiology after 2020: patterns of circulation, epidemic timing and duration, and implications for vaccination strategies
Source: Euro Surveill. 2026 May 28;31(21):2500743. doi: 10.2807/1560-7917.ES.2026.31.21.2500743 (PMC13221616; doi:10.2807/1560-7917.ES.2026.31.21.2500743)
Supplement: Supplement [file 25-00743_DelRICCIO_Supplement.pdf]

This supplementary material is hosted by Eurosurveillance as supporting information alongside the article “*Global influenza epidemiology after 2020: patterns of circulation, epidemic timing and duration, and implications for vaccination strategies*”, on behalf of the authors, who remain responsible for the accuracy and appropriateness of the content. The same standards for ethics, copyright, attributions and permissions as for the article apply. Supplements are not edited by Eurosurveillance and the journal is not responsible for the maintenance of any links or email addresses provided therein.

Classified as ECDC NORMAL

Supplementary Figure 1. Typical timing of the primary peak of influenza detections by country, against the latitude of the country centroid. WHO FluNet database, 2021-2025, “non-sentinel”/”not defined” surveillance data.

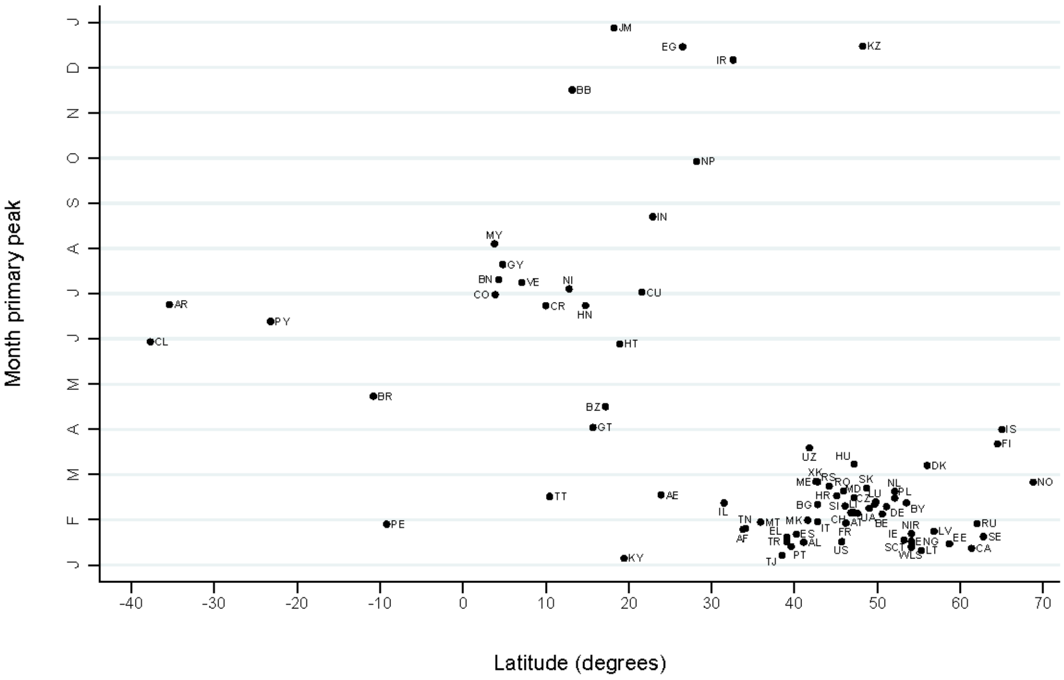

Classified as ECDC NORMAL

Supplementary Figure 2. Duration (in weeks) of influenza epidemics, calculated according to the 75% average annual percentage (75% AAP) method, by latitude. The small grey circles represent individual country-seasons, while the large black circles represent the median values by 10-degree latitude bands (from 40° to 30° south, up to 60° to 70° north); vertical bars indicate the 25th and 75th percentiles (interquartile range) within each latitude band. WHO FluNet database, 2021-2025, “non-sentinel”/”not defined” surveillance data.

Classified as ECDC NORMAL

Supplementary figure 3. Week of epidemic onset, calculated according to the 75% average annual percentage (75% AAP) method, by latitude. The small grey circles represent individual country-seasons, while the large black circles represent the median values by 10-degree latitude bands (from 50° to 40° south, up to 60° to 70° north); vertical bars indicate the 25th and 75th percentiles (interquartile range) within each latitude band. WHO FluNet database, 2021–2025, "sentinel" surveillance data.

Classified as ECDC NORMAL

Supplementary figure 4. Week of epidemic onset, calculated according to the 75% average annual percentage (75% AAP) method, by latitude. The small grey circles represent individual country-seasons, while the large black circles represent the median values by 10-degree latitude bands (from 50° to 40° south, up to 60° to 70° north); vertical bars indicate the 25th and 75th percentiles (interquartile range) within each latitude band. WHO FluNet database, 2021–2025, “non-sentinel”/”not defined” surveillance data.

Classified as ECDC NORMAL

Supplementary Table 1. Global circulation of type A and B influenza viruses according to countries' latitude, WHO region, and season. WHO FluNet database, 2021-2025, "non-sentinel"/"not defined" surveillance data. Only country-seasons with ≥50 reported influenza detections were included.

	N. country-seasons (≥50 cases)	N influenza cases	Influenza type A		Influenza type B		Median cases per season	Median % A	Season with % A			
			N	%	N	%			≥ 80%	≥ 50% to < 80%	≥ 20% to < 50%	< 20%
Geographical area												
Northern hemisphere	224	3,644,765	3,026,598	83.0%	618,167	17.0%	1,685	85.6%	140	73	10	1
Inter-tropical belt	112	155,249	118,987	76.6%	36,262	23.4%	323	79.9%	56	42	12	2
Southern hemisphere	11	130,748	105,453	80.7%	25,295	19.3%	13,719	82.2%	6	5	0	0
WHO Region												
African region	39	10,415	7,781	74.7%	2,634	25.3%	224	77.2%	18	16	4	1
Eastern Mediterranean region	30	92,485	74,141	80.2%	18,344	19.8%	1,232	80.6%	16	11	3	0
European region	168	1,338,983	1,098,092	82.0%	240,891	18.0%	1,802	87.8%	111	50	7	0
Region of the Americas	57	1,815,025	1,551,761	85.5%	263,264	14.5%	599	82.3%	31	20	5	1
South-East Asia region	19	14,124	10,803	76.5%	3,321	23.5%	575	76.7%	8	11	0	0
Western Pacific region	34	659,730	508,460	77.1%	151,270	22.9%	645	83.1%	18	12	3	1
Season												
2021	78	353,558	298,603	84.5%	54,955	15.5%	314	98.0%	62	10	4	2
2022	87	1,014,712	900,264	88.7%	114,448	11.3%	810	80.1%	44	39	4	0
2023	90	1,129,242	853,646	75.6%	275,596	24.4%	938	88.3%	57	24	8	1
2024	92	1,433,250	1,198,525	83.6%	234,725	16.4%	1,215	78.2%	39	47	6	0
**Total**	**347**	**3,930,762**	**3,251,038**	**82.7%**	**679,724**	**17.3%**	**734**	**83.9%**	**202 (58.2%)**	**120 (34.6%)**	**22 (6.3%)**	**3 (0.9%)**

Classified as ECDC NORMAL

Supplementary Table 2. Global circulation of the different influenza virus types, subtypes, and lineages according to season. WHO FluNet database, 2021-2025, “non-sentinel”/”not defined” surveillance data. Only country-seasons with ≥50 reported influenza detections were included.

**Season**	**N season (≥50 cases)**	**N influenza cases**	**A(H3N2)**		**A(H1N1)**		**A other or unsubtyped**		**B Victoria**		**B uncharacterized [(a)]**	
			**N**	**%**	**N**	**%**	**N**	**%**	**N**	**%**	**N**	**%**
2021	78	353,558	50,254	14.2%	5,073	1.4%	243,260	68.8%	48,603	13.7%	6,352	1.8%
2022	87	1,014,712	128,177	12.6%	125,047	12.3%	647,033	63.8%	9,895	1.0%	104,553	10.3%
2023	90	1,129,242	196,311	17.4%	112,789	10.0%	544,504	48.2%	96,876	8.6%	178,720	15.8%
2024	92	1,433,250	74,594	5.2%	231,467	16.1%	892,445	62.3%	17,826	1.2%	216,899	15.1%
**Total**	**347**	**3,930,762**	**449,336**	**11.4%**	**474,376**	**12.1%**	**2,327,242**	**59.2%**	**173,200**	**4.4%**	**506,524**	**12.9%**

[(a)] This also includes 6 influenza B Yamagata detections

Classified as ECDC NORMAL

Supplementary Table 3. Primary peak timing by country (data underlying Figure 1 and Supplementary Figure 1)

Country	Country code	Latitude	Longitude	SENTINEL DATA		NON-SENTINEL/NOT-DEFINED DATA	
Afghanistan	AF	33.8	66.0	11.8	Dec	0.8	Jan
Albania	AL	41.1	20.0	1.3	Feb	0.5	Jan
United Arab Emirates	AE	23.9	54.3	10.6	Nov	1.5	Feb
Argentina	AR	-35.4	-65.2	10.0	Oct-Nov	5.8	Jun
Armenia	AM	40.3	44.9	1.3	Feb		
Austria	AT	47.6	14.1	1.3	Feb	1.1	Feb
Belgium	BE	50.6	4.6	1.2	Feb	1.1	Feb
Burkina Faso	BF	12.3	-1.8	9.5	Oct		
Bangladesh	BD	23.9	90.2	6.5	Jul		
Bulgaria	BG	42.8	25.2	1.1	Feb	1.3	Feb
Bahrain	BH	26.0	50.5	8.9	Sep		
Belarus	BY	53.5	28.0	0.6	Jan	1.4	Feb
Belize	BZ	17.2	-88.7	1.6	Feb	3.5	Apr
Bolivia, Plurinational State of	BO	-16.7	-64.7	4.2	May		
Brazil	BR	-10.8	-53.1	2.7	Mar	3.7	Apr

Classified as ECDC NORMAL

Barbados	BB	13.2	-59.6			10.5	Nov
Brunei Darussalam	BN	4.3	114.4	10.2	Nov	6.3	Jul
Canada	CA	61.4	-98.3			0.4	Jan
Switzerland	CH	46.8	8.2	1.7	Feb	1.2	Feb
Chile	CL	-37.7	-71.4	4.8	May	4.9	May
Cameroon	CM	5.7	12.7	10.0	Oct-Nov		
Congo, The Democratic Republic of the	CD	-2.9	23.6	0.0	Dec-Jan		
Colombia	CO	3.9	-73.1	5.7	Jun	6.0	Jun-Jul
Costa Rica	CR	10.0	-84.2	5.7	Jun	5.7	Jun
Cuba	CU	21.6	-79.0	2.2	Mar	6.0	Jun-Jul
Cayman Islands	KY	19.4	-80.9			0.1	Jan
Czechia	CZ	49.7	15.3	1.5	Feb	1.3	Feb
Germany	DE	51.1	10.4	1.5	Feb	1.3	Feb
Denmark	DK	56.0	10.0	2.3	Mar	2.2	Mar
Dominican Republic	DO	18.9	-70.5	10.6	Nov		
Ecuador	EC	-1.4	-78.8	11.5	Dec		
Egypt	EG	26.5	29.9	11.4	Dec	11.5	Dec

Classified as ECDC NORMAL

England	ENG	54.1	-2.9	3.6	Apr	0.5	Jan
Spain	ES	40.2	-3.6	1.2	Feb	0.7	Jan
Estonia	EE	58.7	25.5	0.0	Dec-Jan	0.5	Jan
Ethiopia	ET	8.6	39.6	11.6	Dec		
Finland	FI	64.5	26.3	4.5	May	2.7	Mar
France	FR	46.2	2.5	1.1	Feb	0.9	Jan
Georgia	GE	42.2	43.5	0.9	Jan		
Ghana	GH	8.0	-1.2	5.5	Jun		
Guinea	GN	10.4	-10.9	7.4	Aug		
Greece	EL	39.1	23.0	2.7	Mar	0.6	Jan
Guatemala	GT	15.7	-90.4	2.1	Mar	3.0	Mar-Apr
Guyana	GY	4.8	-59.0			6.6	Jul
Honduras	HN	14.8	-86.6	5.7	Jun	5.7	Jun
Croatia	HR	45.1	16.4			1.5	Feb
Haiti	HT	18.9	-72.7	0.6	Jan	4.9	May
Hungary	HU	47.2	19.4	1.9	Feb	2.2	Mar
Indonesia	ID	-2.2	117.2	9.6	Oct		

Classified as ECDC NORMAL

India	IN	22.9	79.6	8.2	Sep	7.7	Aug
Ireland	IE	53.2	-8.1	0.7	Jan	0.6	Jan
Iran, Islamic Republic of	IR	32.6	54.3	0.4	Jan	11.2	Dec
Iceland	IS	65.0	-18.6			3.0	Mar-Apr
Israel	IL	31.5	35.0	0.9	Jan	1.4	Feb
Italy	IT	42.8	12.1	1.1	Feb	1.0	Jan-Feb
Jamaica	JM	18.2	-77.3	4.0	Apr-May	11.9	Dec
Jordan	JO	31.2	36.8	11.8	Dec		
Japan	JP	37.6	138.0	0.9	Jan		
Kazakhstan	KZ	48.2	67.3	11.5	Dec	11.5	Dec
Kenya	KE	0.6	37.8	5.9	Jun		
Kyrgyzstan	KG	41.5	74.5	11.8	Dec		
Korea, Republic of	KR	36.4	127.8	0.3	Jan		
Kuwait	KW	29.3	47.6	12.0	Dec-Jan		
Lao People's Democratic Republic	LA	18.5	103.7	9.7	Oct		
Lebanon	LB	33.9	35.9	0.3	Jan		
Libya	LY	25.0	17.0	0.2	Jan		

Classified as ECDC NORMAL

Saint Lucia	LC	13.9	-61.0	10.7	Nov		
Liechtenstein	LI	47.2	9.3			1.2	Feb
Sri Lanka	LK	7.6	80.7	5.1	Jun		
Lithuania	LT	55.3	23.9	1.2	Feb	0.3	Jan
Luxembourg	LU	49.8	6.1	1.6	Feb	1.4	Feb
Latvia	LV	56.9	24.9			0.7	Jan
Morocco	MA	29.8	-8.5	0.8	Jan		
Moldova, Republic of	MD	47.2	28.5	1.3	Feb	1.5	Feb
Madagascar	MG	-19.4	46.7	11.6	Dec		
Maldives	MV	3.7	73.5	9.7	Oct		
Mexico	MX	23.9	-102.5	0.1	Jan		
North Macedonia	MK	41.6	21.7	1.3	Feb	1.0	Jan-Feb
Malta	MT	35.9	14.4			1.0	Jan-Feb
Myanmar	MM	21.2	96.5	7.5	Aug		
Montenegro	MNE	42.8	19.2	1.7	Feb	1.8	Feb
Mozambique	MZ	-17.3	35.5	1.9	Feb		
Malaysia	MY	3.8	109.7	6.2	Jul	7.1	Aug

Classified as ECDC NORMAL

Namibia	NA	-22.0	17.0	6.3	Jul		
New Caledonia	NC	-21.3	165.7	3.4	Apr		
Niger	NE	17.4	9.4	1.5	Feb		
Nicaragua	NI	12.8	-85.0	6.0	Jun-Jul	6.1	Jul
Northern Ireland	NIR	54.1	-2.9	0.9	Jan	0.7	Jan
Netherlands	NL	52.1	5.3	1.4	Feb	1.6	Feb
Norway	NO	68.8	15.3	1.9	Feb	1.8	Feb
Nepal	NP	28.2	83.9	8.5	Sep	8.9	Sep
New Zealand	NZ	-41.8	171.5	7.2	Aug		
Panama	PA	8.5	-80.1	5.8	Jun		
Peru	PE	-9.2	-74.4	0.9	Jan	0.9	Jan
Poland	PL	52.1	19.4	1.6	Feb	1.5	Feb
Portugal	PT	39.6	-8.5	11.8	Dec	0.4	Jan
Paraguay	PY	-23.2	-58.4	5.5	Jun	5.4	Jun
Palestine, State of	PS	31.9	35.2	11.7	Dec		
Romania	RO	45.9	25.0	1.5	Feb	1.6	Feb
Russian Federation	RU	62.0	96.7	0.2	Jan	0.9	Jan

Classified as ECDC NORMAL

Rwanda	RW	-2.0	29.9	1.2	Feb		
Saudi Arabia	SA	24.1	44.5	10.3	Nov		
Scotland	SCT	54.1	-2.9	0.5	Jan	0.4	Jan
Senegal	SN	14.4	-14.5	8.7	Sep		
El Salvador	SV	13.7	-88.9	5.9	Jun		
Somalia	SO	10.0	49.0	4.1	May		
Serbia	RS	44.2	20.8	1.6	Feb	1.7	Feb
South Sudan	SS	7.3	30.2	7.0	Jul-Aug		
Suriname	SR	4.1	-55.9	3.6	Apr		
Slovakia	SK	48.7	19.5	1.6	Feb	1.7	Feb
Slovenia	SI	46.1	14.8	1.5	Feb	1.3	Feb
Sweden	SE	62.8	16.7	1.8	Feb	0.6	Jan
Syrian Arab Republic	SY	35.0	38.5	12.0	Dec-Jan		
Togo	TG	8.5	1.0	9.5	Oct		
Tajikistan	TJ	38.5	71.0	12.0	Dec-Jan	0.2	Jan
Timor-Leste	TL	-8.8	125.8	1.7	Feb		
Trinidad and Tobago	TT	10.5	-61.3			1.5	Feb

Classified as ECDC NORMAL

Tunisia	TN	34.1	9.6	1.2	Feb	0.8	Jan
Turkey	TR	39.1	35.2	0.8	Jan	0.5	Jan
Uganda	UG	1.3	32.4	3.1	Apr		
Ukraine	UA	49.0	31.4	1.8	Feb	1.3	Feb
Uruguay	UY	-32.8	-56.0	5.6	Jun		
United States	US	45.7	-112.5	1.0	Jan-Feb	0.5	Jan
Uzbekistan	UZ	41.8	63.1	11.1	Dec	2.6	Mar
Saint Vincent and the Grenadines	VC	13.2	-61.2	4.8	May		
Venezuela, Bolivarian Republic of	VE	7.1	-66.2	8.8	Sep	6.2	Jul
Viet Nam	VN	16.6	106.3	0.1	Jan		
Wales	WLS	54.1	-2.9	0.9	Jan	0.4	Jan
Kosovo	XK	42.6	20.9	1.0	Jan-Feb	1.8	Feb
South Africa	ZA	-29.0	25.1	5.2	Jun		

51

Classified as ECDC NORMAL
